# Supplementary material for: Specific Binding of the α-Component of the Lantibiotic Lichenicidin to the Peptidoglycan Precursor Lipid II Predetermines Its Antimicrobial Activity
Source: Int J Mol Sci. 2023 Jan 10;24(2):1332. doi: 10.3390/ijms24021332 (PMC9863751; doi:10.3390/ijms24021332)
Supplement: Supplementary file 1 [file ijms-24-01332-s001.zip › ijms-2048484-supplementary.pdf]

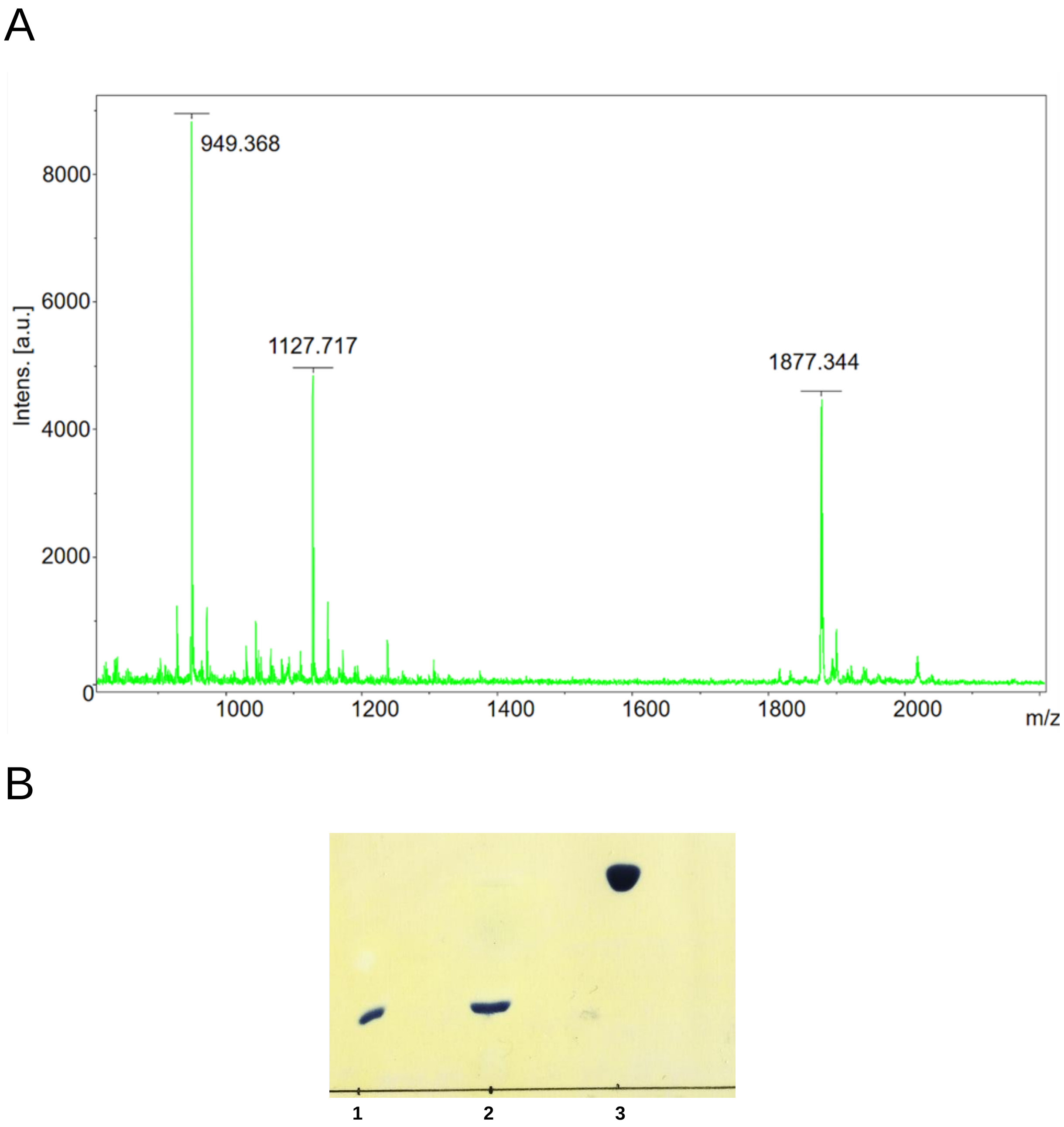

Figure S1. (A) Typical mass spectrometry of purified lipid II. Final preparation containing lipid II (m/z 1877), and the by-products: GlcNAc-MurNAc-pentapeptide (m/z 949) and GlcNAc-MurNAc-(pentapeptide)-pyrophosphate (m/z 1127). **(B) Thin layer chromatography of purified lipid II** (2) in comparison to the following control samples: lipid II control (1) and C55P precursor (3).

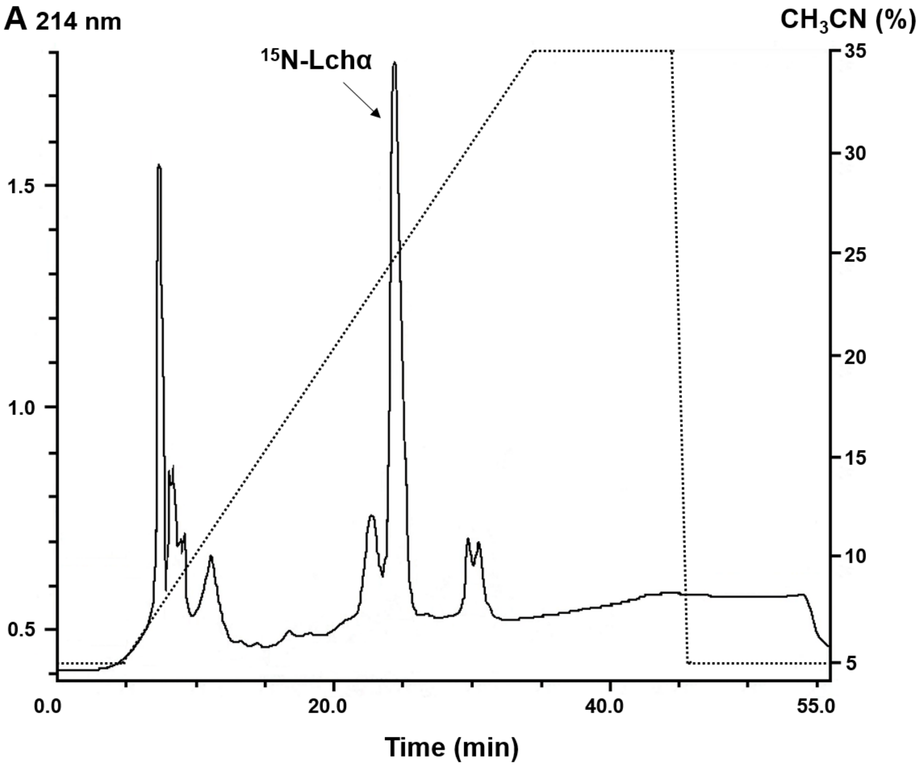

Figure S2. Reversed-phase HPLC purification of ^{15}N-Lchα.

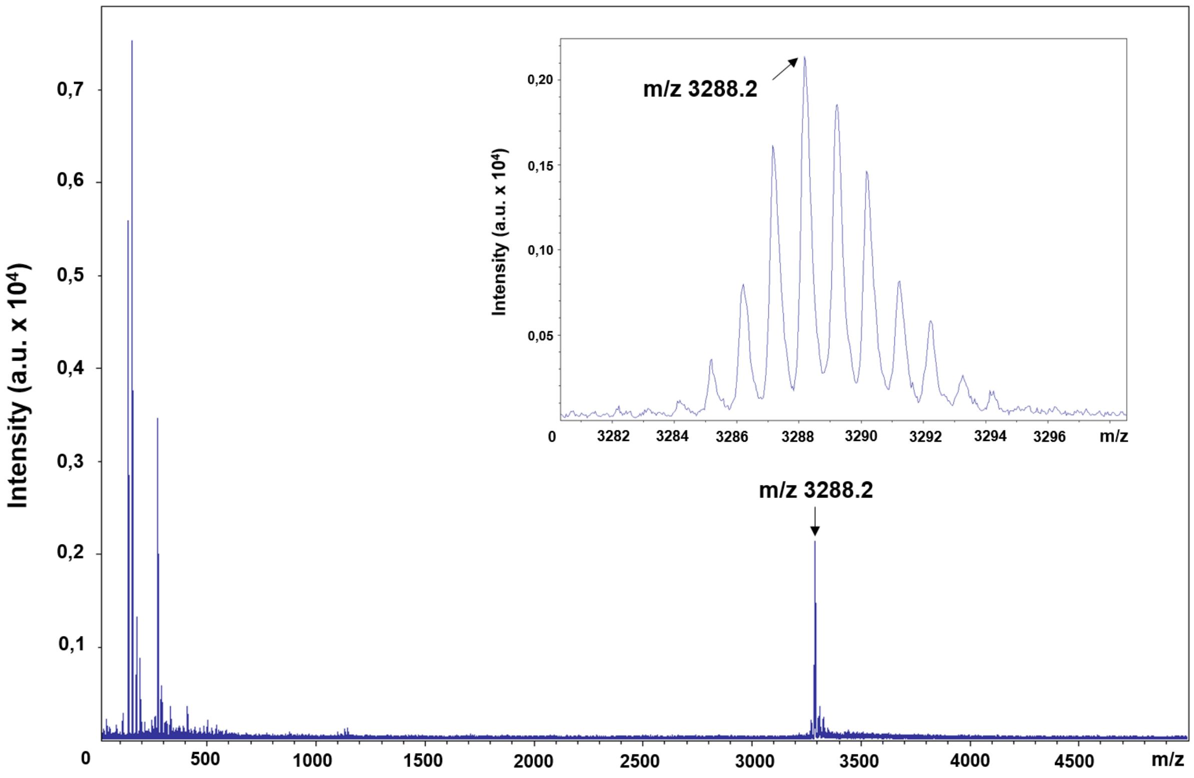

Figure S3. **MALDI-TOF mass-spectrum of ^{15}N-labeled Lchα.** The calculated m/z $[M+H]^+$ for fully ^{15}N-Lchα is 3288.6.

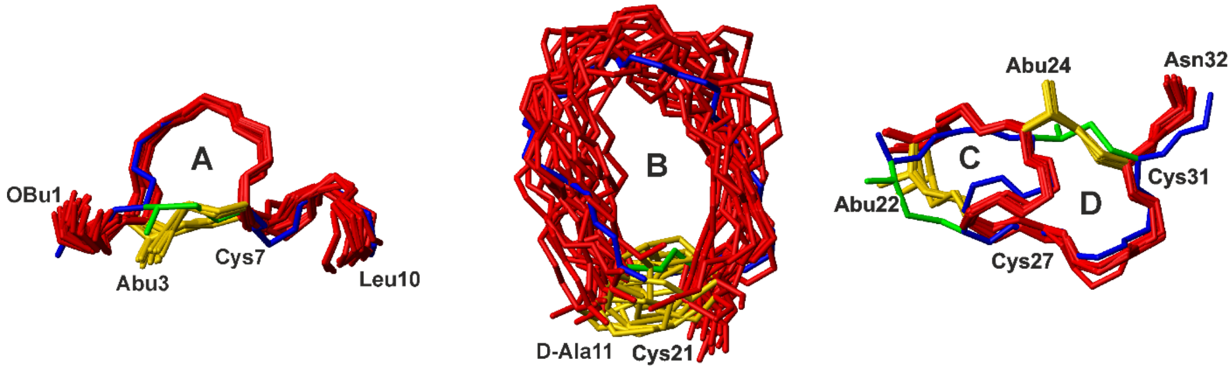

Figure S4. Superimposition of 20 calculated structures of Lchα at the level of individual domains. The peptide backbone is colored *red*. The lanthionine and methyllanthionine bridges are colored *orange*. Thioether bridging rings are marked with the capital letters A, B, C, and D. The previous structure of Lchα with incorrect configuration at the Cβ atom of Abu residues (R instead of S) is shown by *blue/green*.

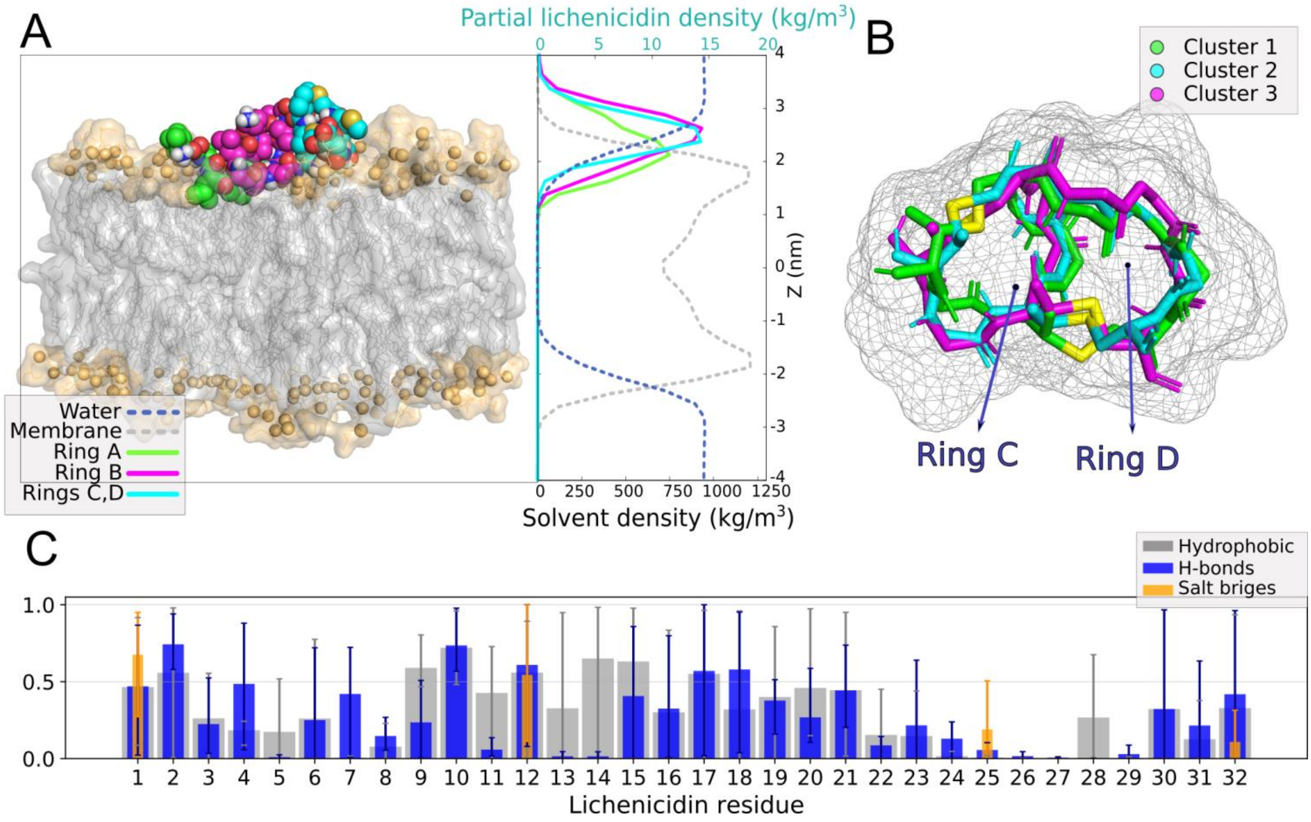

Figure S5. Lchα interaction with the model bacterial membrane, revealed in MD simulations. (A) Overview of Lchα interaction with the membrane (*left*). The average density profiles of Lchα rings A (*green*), B (*magenta*) and C, D (*cyan*) (top axis) at the membrane-water interface (bottom axis) as a function of the distance from the bilayer center (Z) (*right*). **(B)** The membrane-bound states of the Lchα *C*-terminal fragment (rings C and D, residues 22–32) superimposed over the backbone atoms. Disordered *C*-terminal residues and side chains are not shown for clarity. Clusters of conformers are numbered as their population decreases. Molecular surface of the corresponding structures in clusters ##1-4 with the side chains is shown with grey mesh. **(C)** Lchα–membrane interaction profile. Patterns of hydrophobic (*grey*) H-bonds (*blue*) and ionic (*orange*) protein–lipid interactions are shown (distance cutoff of 4 Å). Error bars indicate the deviation along the trajectories. Please note, that the *N*-terminal 2-oxobutyryl group was replaced with the valine residue.

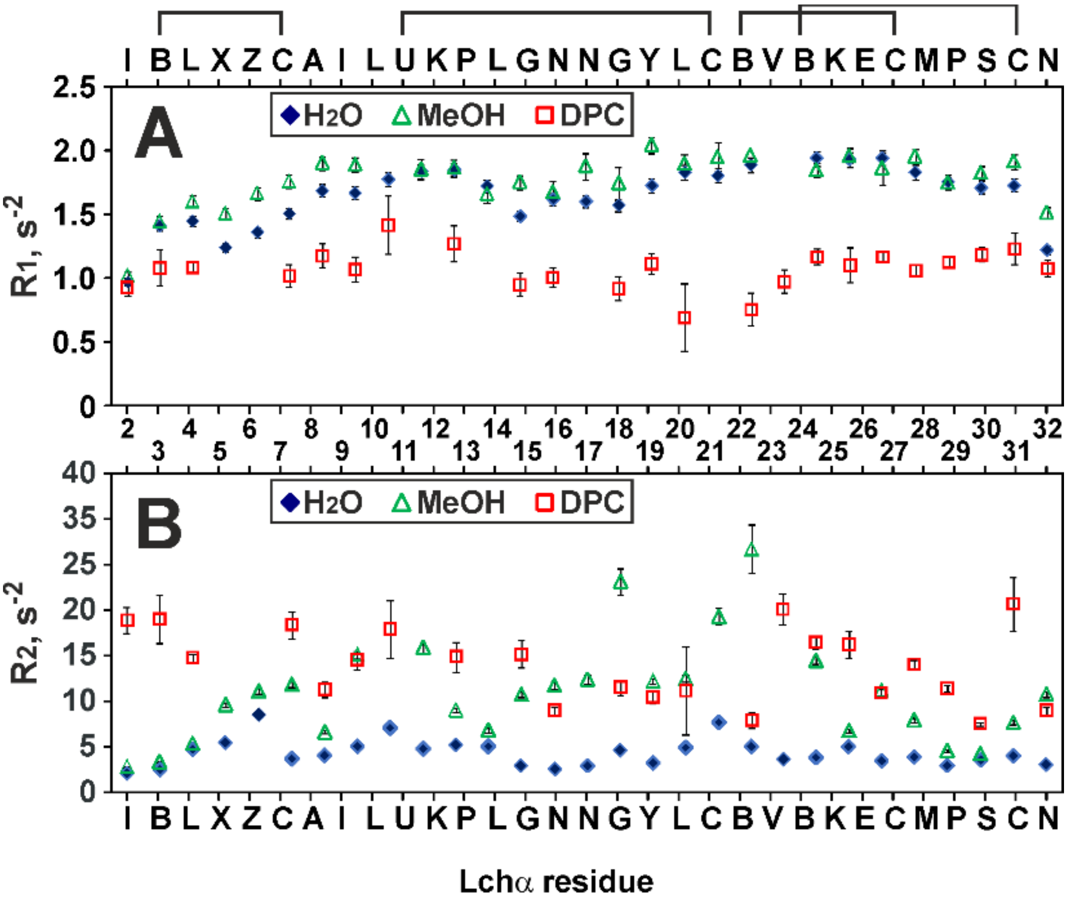

Figure S6. R1 and R2 ^{15}N relaxation rates measured at 80 MHz for Lchα in water, methanol, and DPC micelles.

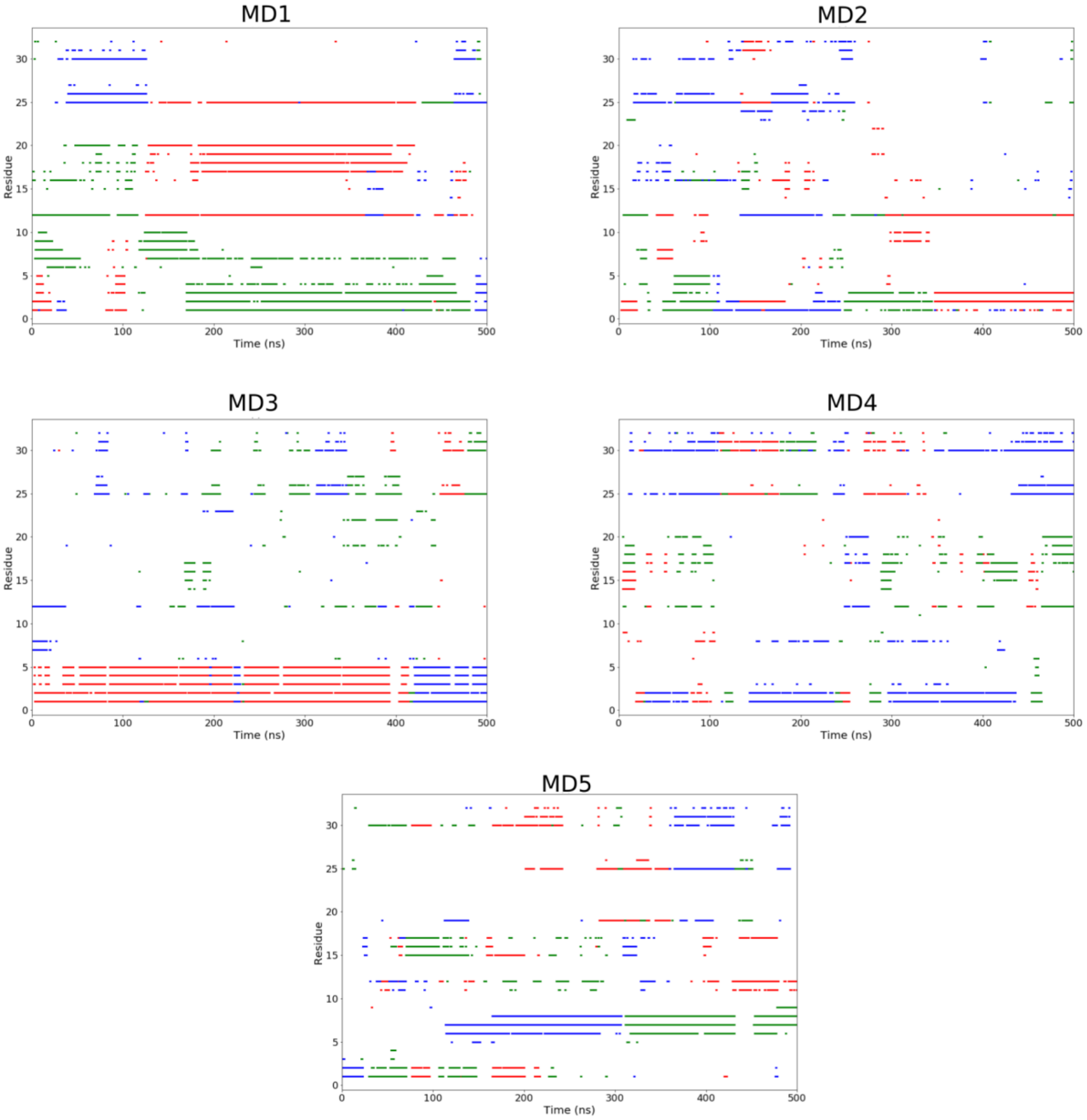

Figure S7. Spontaneous formation of Lchα/DMPPi complexes in MD. Hydrogen bonds maps between the peptide backbone and three DMPPi ions. Each dot indicates the H-bond between different DMPPi ions (*different colors*) and particular residue of Lchα (vertical axis) at a given MD time (horizontal axis). Results of five independent MD simulations.

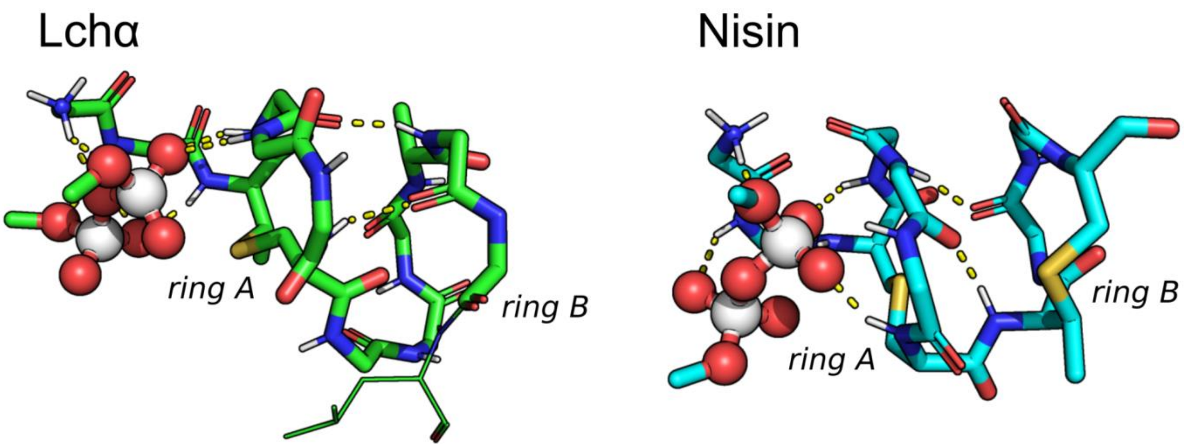

Figure S8. Binding modes of Lchα's and nisin's *N*-terminus to DMPPi revealed in MD simulations. Backbone of the peptides residues 1–13 is shown with *sticks*, wherein carbon — *green* (Lchα) or *cyan* (nisin), oxygen — *red*, phosphorus — *white*, sulphur — *yellow*; peptides side chain atoms are hidden. H-bonds are depicted with yellow dotted lines.

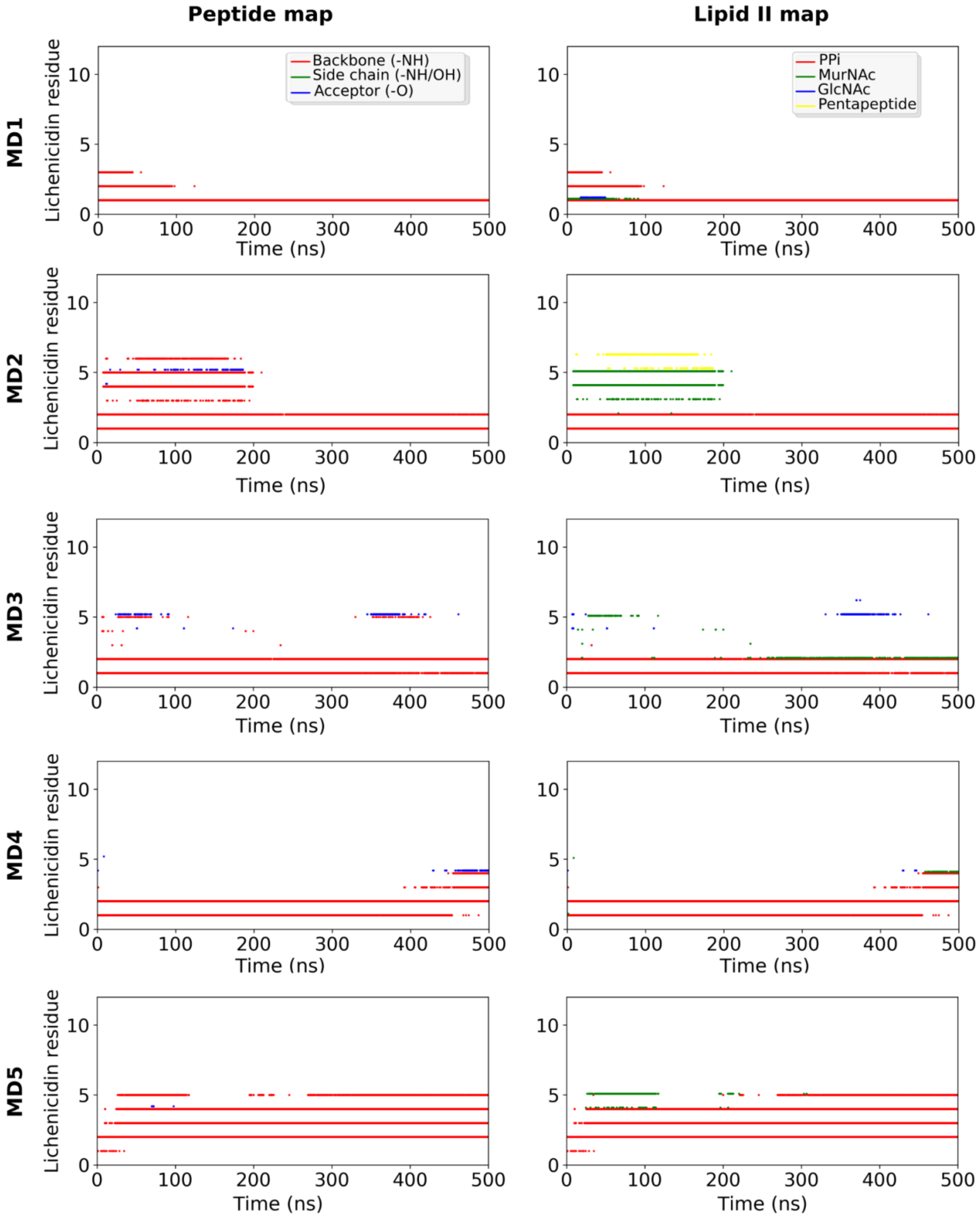

Figure S9. Intermolecular hydrogen bonds in the *N*-terminal Lch*α* / lipid II complexes during MD simulations. Hydrogen bonding maps demonstrate organization and dynamics of the complexes. *Left panel:* peptide's groups taking part in the h-bond formation in a certain MD time: backbone amide group which donates the proton (*red dots*), side chains (*green*) and backbone carbonyl group which accepts the proton (*blue*). *Right panel:* lipid II's groups participating in the formation of the same h-bonds: PPi (*red*), MurNAc sugar (*green*), GlcNAc sugar (*blue*) and the pentapeptide (*yellow*).

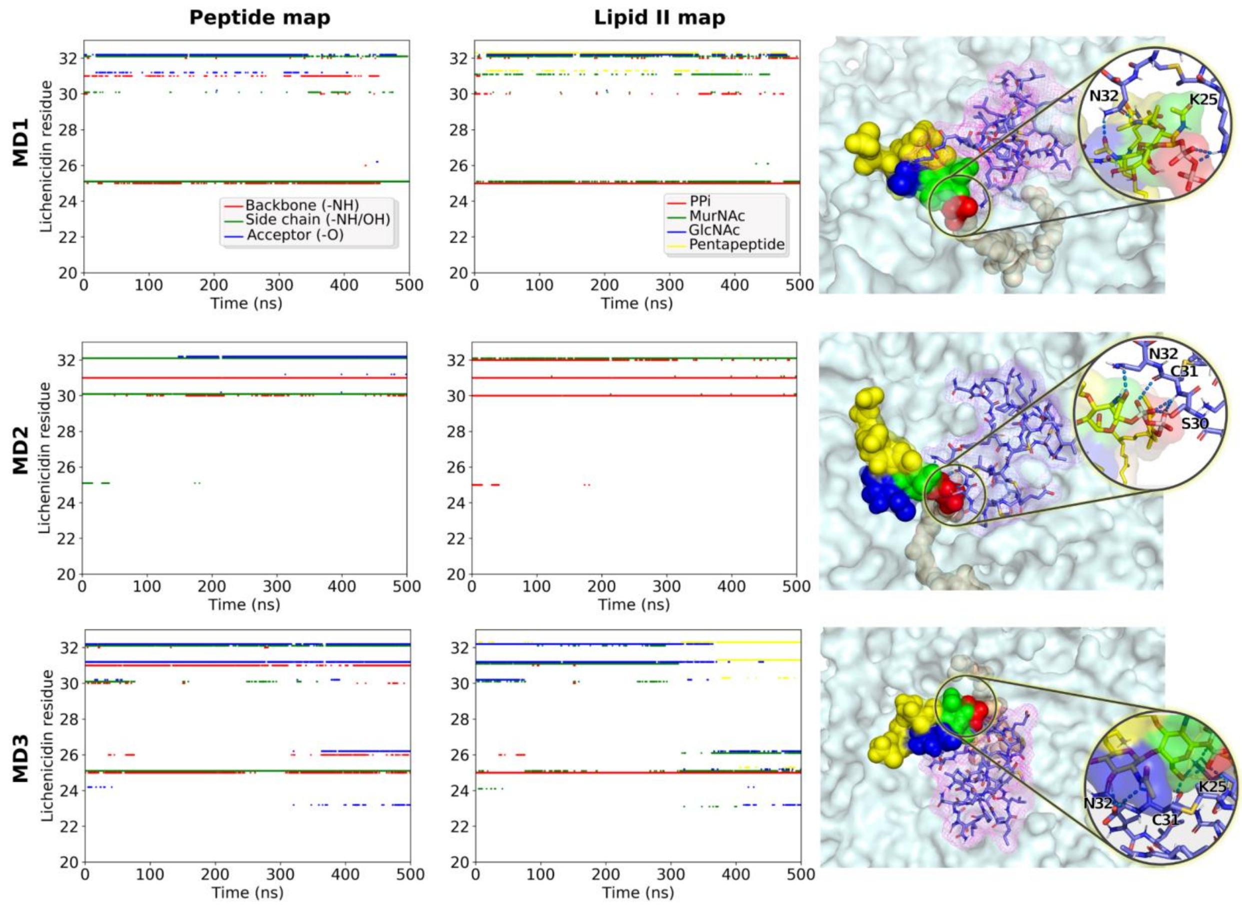

Figure S10. Intermolecular hydrogen bonds in *C*-terminal Lchα / lipid II complexes in the membrane during MD simulations. Hydrogen bonding maps demonstrate organization and dynamics of the complexes. *Left panel:* peptide's groups taking part in the h-bond formation in a certain MD time: backbone amide group which donates the proton (*red dots*), side chains (*green*) and backbone carbonyl group which accepts the proton (*blue*). *Middle panel:* lipid II's groups participating in the formation of the same h-bonds: PPi (*red*), MurNAc sugar (*green*), GlcNAc sugar (*blue*) and the pentapeptide (*yellow*). *Right panel:* the representative snapshots from MD. Lipid II is color-coded as in the *middle panel*. The surface of the membrane is shown. Interactions are zoomed in the *insets*.

Table S1. Chemical shift values (ppm) of the major Lch*α* form in water (30 °C, pH 4.0)

Res	atom	δ_H	$\delta_C(\delta_N)$	Res	atom	δ_H	$\delta_C(\delta_N)$	Res	atom	δ_H	$\delta_C(\delta_N)$
**Obu1**	**3**	2.83	30.2	**Lys12**	H^N	8.29	123.0	**Abu22**	H^N	8.12	115.3
	**4**	1.02	6.3		**α**	4.63			**α**	4.60	
**Ile2**	H^N	8.54	117.5		**β**	1.80/ 1.70	29.9		**β**	3.69	44.4
	**α**	4.27	58.7		**γ**	1.42/ 1.37	22.0		**γ**	1.31	19.8
	**β**	1.89	35.8		**δ**	1.64	26.5	**Val23**	H^N	7.51	119.2
	γ_1	1.48/1.19	24.9		**ε**	2.94	39.4		**α**	4.50	
	γ_2	0.91	14.9	**Pro13**	**α**	4.30			**β**	2.00	32.0
	**δ**	0.84	10.1		**β**	2.14/ 1.87	29.1		γ_1	0.87	18.7
**Abu3**	H^N	8.44	117.4		**γ**	2.00/ 1.92	24.7		γ_2	0.76	17.6
	**α**	4.58			**δ**	3.73/ 3.61	47.9	**Abu24**	H^N	7.95	117.3
	**β**	3.52	41.0		H^N	8.12	121.2		**α**	4.64	
	**γ**	1.32	19.1	**Leu14**	**α**	4.32			**β**	3.66	42.1
**Leu4**	H^N	7.85	122.3		**β**	1.54	39.6		**γ**	1.26	19.6
	**α**	4.41			**γ**	1.48	24.3	**Lys25**	H^N	8.85	122.0
	**β**	1.69/1.64	38.4		**δ**	0.85/ 0.82	22.2/ 20.8		**α**	4.28	
	**γ**	1.41	24.2	**Gly15**	H^N	8.22	109.6		**β**	1.82	29.9
	**δ**	0.78/0.78	22.3/20.3		**α**	3.96/ 3.80	42.8		**γ**	1.48	22.8
**Dha5**	H^N	9.38	126.8	**Asn16**	H^N	8.35	119.2		**δ**	1.70	26.5
	**β**	5.88/5.70			**α**	4.65			**ε**	3.03	39.4
**Dhb6**	H^N	9.84	119.1		**β**	2.83/ 2.70	36.1	**Glu26**	H^N	8.50	116.2
	**β**	6.66			H^{δ}	7.49/ 6.85	112.6		**α**	4.02	55.6
	**γ**	1.79	12.8	**Asn17**	H^N	8.34	117.6		**β**	2.05	25.6
**Cys7**	H^N	7.98	116.0		**α**	4.62			**γ**	2.43	31.7
	**α**	4.32			**β**	2.82/ 2.72	36.1	**Cys27**	H^N	7.12	113.2
	**β**	3.07	30.8		H^{δ}	7.48/ 6.85	112.6		**α**	4.66	
**Ala8**	H^N	8.14	123.7	**Gly18**	H^N	8.17	108.1		**β**	2.98/2.78	
	**α**	4.22	50.6		**α**	3.85	42.8	**Met28**	H^N	7.87	119.3
	**β**	1.38		**Tyr19**	H^N	7.91	119.9		**α**	4.92	
**Ile9**	H^N	7.71	117.9		**α**	4.56			**β**	2.01	32.1
	**α**	4.04	58.5		**β**	2.94/2.88			**γ**	2.48	29.2

	**β**	1.79			**δ**	6.99			**E**	2.12	14.5
	**γ_1**	1.38/1.10	24.6		**ε**	6.77		**Pro29**	**α**	4.33	
	**γ_2**	0.80	14.9	**Leu20**	**H^N**	8.15	123.0		**β**	2.32/ 1.94	29.3
	**δ**	0.78	10.3		**α**	4.36			**γ**	2.04/2.00	24.6
**Leu10**	**H^N**	7.99	124.6		**β**	1.51/ 1.48	40.4		**δ**	3.80/ 3.76	48.2
	**α**	4.27			**γ**	1.43	24.3	**Ser30**	**H^N**	7.60	109.5
	**β**	1.57/1.45	39.8		**δ**	0.85/ 0.80	22.2/20.9		**α**	4.31	
	**γ**	1.54	24.3	**Cys21**	**H^N**	8.42	122.0		**β**	3.93/ 3.77	60.2
	**δ**	0.84/0.80	22.3/20.9		**α**	4.61		**Cys31**	**H^N**	7.73	122.6
**D-Ala11**	**H^N**	8.17	118.9		**β**	2.88	31.0		**α**	4.23	
	**α**	4.53							**β**	2.73/ 2.89	32.8
	**β**	3.13/2.90						**Asn32**	**H^N**	8.11	127.9
									**α**	4.53	
									**β**	2.74/ 2.64	38.0
									**H^δ**	7.40/ 6.81	113.0

Table S2. Chemical shift values (ppm) of the major Lch*α* form in methanol (27 °C, pH 3.5)

Res	atom	δ_H	$\delta_C(\delta_N)$	Res	atom	δ_H	$\delta_C(\delta_N)$	Res	atom	δ_H	$\delta_C(\delta_N)$
**Ile2**	H^N	8.43	114.5	**Lys12**	H^N	8.20	120.1	**Val23**	H^N	7.73	119.1
	α	4.23	58.8		α	4.65			α	4.46	58.8
	β	1.62	24.6		β	1.89/1.82	26.3		β	2.13	30.9
	γ_1	1.94/1.24	24.9		γ	1.52/1.46			γ_1	0.94	18.7
	γ_2	0.98	14.6		δ	1.71	26.6		γ_2	0.86	17.9
	δ	0.94	9.7	**Pro13**	α	4.41	61.5	**Abu24**	H^N	8.01	116.7
**Abu3**	H^N	8.27	115.2		β	2.17/1.92	28.9		α	4.75	50.5
	α	4.71	57.6		γ	2.08/1.95	24.8		β	3.77	71.2
	β	3.70			δ	3.77/3.69	47.4		γ	1.27	31.6
	γ	1.32	18.6	**Leu14**	H^N	8.12	117.1	**Lys25**	H^N	8.93	122.2
**Leu4**	H^N	7.78	120.6		α	4.28	52.8		α	4.40	55.3
	α	4.55	52.1		β	1.72/1.64			β	1.92/1.82	30.3
	β	1.81	26.4		γ	1.68	20.4		γ	1.66	23.0
	γ	1.75/1.61	24.4/15.86		δ	0.91			δ	3.14/3.06	39.4
	δ	0.89	22.0	**Gly15**	H^N	8.30	106.9	**Glu26**	H^N	8.51	113.7
**Dha5**	H^N	9.65	126.2		α	3.97/3.87	42.9		α	4.09	55.2
	β	5.56 /4.96		**Asn16**	H^N	8.22	118.0		β	2.08	
**Dhb6**	H^N	10.13	117.4		α	4.76	57.1		γ	2.57	30.4
	β	6.66			β	2.94/2.67	36.4	**Cys27**	H^N	7.21	112.2
	γ	1.87	11.86	**Asn17**	H^N	8.26	116.2		α	4.75	54.8
**Cys7**	H^N	8.03	115.5		α	4.68	50.7		β	3.00	39.7
	α	4.26			β	2.85/2.91	37.3	**Met28**	H^N	7.81	116.9
	β	3.17		**Gly18**	H^N	8.2	105.2		α	5.01	50.3
**Ala8**	H^N	8.23	121.9		α	3.93/3.74	42.6		β	2.04	
	α	4.21	51.7	**Tyr19**	H^N	7.95	118.1		γ	2.52	29.6
	β	1.61			α	4.52	52.2	**Pro29**	α	4.38	62.7
**Ile9**	H^N	7.47	112.3		β	3.03/2.90	36.5		β	2.37/1.99	29.2
	α	4.14	65.1		δ	7.07			γ	2.10	24.6
	β	1.96			ε	6.69			δ	3.94/3.83	48.0
	γ_1	1.51/1.26	24.9	**Leu20**	H^N	8.12	118.98	**Ser30**	H^N	7.27	106.3

	**γ_2**	0.93	14.7		**α**	4.40			**α**	4.24	55.2
	**δ**	0.85	6.8		**β**	1.61			**β**	4.05/3.72	60.2
**Leu10**	**H^N**	7.73	119.1		**γ**	0.91		**Cys31**	**H^N**	7.55	121.4
	**α**	4.30	52.8	**Cys21**	**H^N**	8.26	120.2		**α**	4.17	55.3
	**β**	1.74/1.57			**α**	4.50			**β**	2.83/2.92	31.7
	**γ**	1.77			**β**	2.92	29.8	**Asn32**	**H^N**	8.37	124.7
	**δ**	0.94/0.91	22.2	**Abu22**	**H^N**	7.83	113.8		**α**	4.91	49.7
**D-Ala11**	**H^N**	8.23	117.4		**α**	4.69	58.4		**β**	2.81/2.68	37.4
	**α**	4.52			**β**	3.77	44.9				
	**β**	2.92/3.22	32.9		**γ**	1.35					

Table S3. Chemical shift values (ppm) of the major Lchα form in DPC micelles (45 °C, pH 5.8)

Res	atom	δ_H	$\delta_C(\delta_N)$	Res	atom	δ_H	$\delta_C(\delta_N)$	Res	atom	δ_H	$\delta_C(\delta_N)$
**Obu1**	**3**	1.83	30.2	**Lys12**	**H^N**	8.43	122.3	**Val23**	**H^N**	7.57	117.5
	**4**	2.90	6.8		**α**	4.63			**α**	4.66	
**Ile2**	**H^N**	8.55	116.4		**β**	1.98	30.0		**β**	2.13	27.6
	**α**	4.32			**γ**	1.61	22.6		**γ_1**	1.02	19.0
	**β**	2.07	35.5		**δ**	1.84	26.7		**γ_2**	0.92	17.8
	**γ_1**	1.68/ 1.31	25.1		**ε**	3.18	39.6	**Abu24**	**H^N**	7.75	116.3
	**γ_2**	1.07	15.4	**Pro13**	**α**	4.51			**α**	4.95	
	**δ**	0.97	11.0		**β**	2.32/ 1.97	28.9		**β**	3.88	48.3
**Abu3**	**H^N**	8.35	116.1		**γ**	2.19/ 2.09	25.2		**γ**	1.42	19.8
	**α**	4.81			**δ**	3.83/ 3.78	47.8	**Lys25**	**H^N**	9.08	122.2
	**β**	3.80		**Leu14**	**H^N**	7.97	118.0		**α**	4.23	
	**γ**	1.46	18.4		**α**	4.49			**β**	1.96	30.0
**Leu4**	**H^N**	7.95	122.3		**β**	1.77			**γ**	1.52/ 1.58	22.4
	**α**	4.54			**γ**				**δ**	1.81	24.8
	**β**	1.81	39.5		**δ**				**ε**	3.10	39.7
	**γ**	1.65	25.1	**Gly15**	**H^N**	8.29	108.8	**Glu26**	**H^N**	8.83	116.6
	**δ**	0.96	21.5		**α**	4.14/ 4.03	42.9		**α**	4.15	
**Dha5**	**H^N**	9.16	125.2	**Asn16**	**H^N**	8.54	119.5		**β**	2.17	26.6
	**β**	5.56/ 4.96			**α**	4.78			**γ**	2.44	34.1
**Dhb6**	**H^N**	10.33	116.4		**β**	3.00/ 2.87	36.3	**Cys27**	**H^N**	7.32	113.2
	**β**	6.88			**H^δ**	7.65/ 6.92	112.2		**α**	4.81	
	**γ**	1.95	12.9	**Asn17**	**H^N**	8.30	117.5		**β**	3.05/ 2.91	37.3
**Cys7**	**H^N**	8.20	116.2		**α**	4.82		**Met28**	**H^N**	8.09	118.7
	**α**	4.38			**β**	2.99/ 2.85	37.0		**α**	5.06	
	**β**	3.25	43.7		**H^δ**	7.66/ 6.96	112.7		**β**	2.27	32.4
**Ala8**	**H^N**	8.54	123.0	**Gly18**	**H^N**	8.19	107.9		**γ**	2.68	29.5
	**α**	4.39			**α**	4.03	42.7		**ε**	2.31	15.0
	**β**	1.67	24.5	**Tyr19**	**H^N**	8.09	119.7				
**Ile9**	**H^N**	7.66	113.6		**α**	4.68		**Pro29**	**α**	4.49	62.7
	**α**	4.30			**β**	3.09/ 3.01	36.9		**β**	2.48/ 2.10	

	**β**	2.07	25.1
	**γ_1**	1.35/ 1.64	24.5
	**γ_2**	1.03	15.4
	**δ**	1.00	10.3
**Leu10**	**H^N**	7.83	119.6
	**α**	4.40	
	**β**	1.83/ 1.72	39.9
	**γ**	1.67	29.8
	**δ**	1.01/ 0.98	22.8
**D-Ala11**	**H^N**	8.23	117.3
	**α**	4.45	
	**β**	3.00/ 2.85	

	**δ**	7.17	
	**ε**	6.47	
**Leu20**	**H^N**	8.18	120.1
	**α**	4.49	
	**β**	1.72	40.1
	**γ**	1.64	
	**δ**	1.00/ 0.96	22.6/ 22.5
**Cys21**	**H^N**	8.38	122.9
	**α**	4.54	
	**β**	3.06	
**Abu22**	**H^N**	8.23	112.8
	**α**	4.73	
	**β**	3.85	
	**γ**	1.42	19.9

	**γ**	2.22/ 2.15	24.8
	**δ**	3.93/ 3.88	48.4
**Ser30**	**H^N**	7.88	101.0
	**α**	4.44	
	**β**	4.02/ 3.95	60.5
**Cys31**	**H^N**	7.82	121.2
	**α**	4.45	
	**β**	3.10/ 2.94	37.0
**Asn32**	**H^N**	8.01	127.2
	**α**	4.62	
	**β**	2.89/ 2.78	38.6
	**H^{δ}**	7.41/ 6.96	112.8

Table S4. Chemical shift values (ppm) of lipid II in DPC micelles (45 °C, pH 5.8)

**Res**	**atom**	δ_H	$\delta_C(\delta_N)$	**Res**	**atom**	δ_H	$\delta_C(\delta_N)$	**Res**	**atom**	δ_H	$\delta_C(\delta_N)$
**Ala1**	H^N	8.30		**GlcNAc**	**H**	8.38		**IR_2**	H_1	2.22	26.3
	**α**	4.51	49.9		H_1	4.77	100.1		H_2	5.34	45.1
	**β**	1.52	16.9		H_2	3.56	70.8		H_4	2.24	32.0
**γ-D-Glu2**	H^N	7.88			H_3	3.56	76.1		H_5	1.83	23.1
	**α**	4.33	54.6		H_4	4.27	78.4	**IR_3**	H_1	2.20	26.3
	**β**	2.47/2.10	32.1		H_5	3.75	74.1		H_2	5.27	45.1
	**γ**	2.26/2.02	28.4		H_6	4.13/3.84	61.5		H_4	2.18	32.2
**Lys3**	H^N	8.29			H_8	2.14	26.4		H_5	1.80	23.2
	**α**	4.38	54.4	**MurNAc**	**H**	8.49		**IR_4**	H_1	2.19	26.3
	**β**	1.96/1.92	30.8		H_1	5.60	94.0		H_2	5.36	45.0
	**γ**	1.61/1.64	22.3		H_2	4.27	72.6		H_4	2.17	32.2
	**δ**	1.84	26.6		H_3	3.99	78.0		H_5	1.78	23.1
	**ε**	3.16	39.7		H_4	4.10	74.4	**IR_5**	H_1	2.18	26.3
**D-Ala4**	H^N	7.97			H_5	3.96	70.1		H_2	5.24	45.0
	**α**	4.44	50.1		H_6	4.04/3.89	60.2		H_4	2.15	32.2
	**β**	1.58	17.3		H_8	1.95	22.3		H_5	1.76	23.1
**D-Ala5**	H^N	7.91			H_{11}	1.59	19.0	**IR_{11}**	H_1	2.12	26.4
	**α**	4.27	51.1	**IR_1**	H_1	4.6	62.5		H_2	5.15	44.2
	**β**	1.48	17.9		H_2	5.61	42.2		H_4	1.70	25.3
					H_4	2.28	32.1		H_5	1.63	15.5
					H_5	1.89	23.0				

Table S5. Statistics for the best CYANA structures of the major conformational state of Lchα in methanol solution

Parameter (Unit)	Quantity	Lchα
No. of structures	calculated	200
	selected	20
Target function (Å^2)		0.62± 0.06
No. of distance constraints	NOE	158/70
(upper/lower)	Lanthionine-bond (4 bonds)	8/4
No. of torsion angle constraints	Angle ϕ	16
Upper constraint violations (>0.2 Å)	No.	1
	max (Å)	0.26
Lower constraint violations (>0.2 Å)	No.	0
Van der Waals constraint violations (>0.2 Å)	No.	0
Angle constraint violations (>5°)	No.	0
rmsd of atomic coordinates (Å)	backbone	5.37 ± 1.79
overall (Obu-Asn32)	heavy atoms	6.25 ± 2.01
rmsd of atomic coordinates (Å)	backbone	0.83 ± 0.47
N-terminal domain (Ile2-Leu10)	heavy atoms	1.22 ± 0.47
rmsd of atomic coordinates (Å)	backbone	2.28 ± 0.48
central domain (D-Ala11-Cys21)	heavy atoms	3.67 ± 0.66
rmsd of atomic coordinates (Å)	backbone	0.49 ± 0.20
C-terminal domain (Abu22-Asn32)	heavy atoms	1.15 ± 0.24
